# BDNF–amyloid-*β* Axis in Alzheimer’s disease: molecular mechanisms and therapeutic perspectives

**DOI:** 10.3389/fnmol.2026.1884003

**Published:** 2026-07-08

**Authors:** Fangfang Liu, Yuanmao Huang, Aiping Wang, Peiyan Liu, Haolin Wu, Haofei Fan

**Affiliations:** 1 Clinical Laboratory, Nanping First Hospital Affiliated to Fujian Medical University, Nanping, China; 2 Faculty of Basic Medical Sciences, Department of Basic Medicine, Hainan Vocational University of Science and Technology, Haikou, China; 3 International Center for Aging and Cancer, Hainan Medical University, Haikou, China

**Keywords:** Alzheimer’s disease, amyloid-*β*, brain-derived neurotrophic factor, neuronal dysfunction, TrkB signaling

## Abstract

Alzheimer’s disease (AD), the most common cause of dementia in older adults, is characterized by progressive cognitive decline, synaptic dysfunction, and neuronal loss. Among the multifactorial mechanisms implicated in AD, reciprocal interactions between brain-derived neurotrophic factor (BDNF) and amyloid-*β* (A*β*) have attracted increasing attention as a convergent axis linking amyloid pathology to impaired neurotrophic support. BDNF promotes neuronal resilience, synaptic plasticity, and cognitive function primarily through the activation of its high-affinity receptor, tropomyosin receptor kinase B (TrkB), and downstream signaling pathways, including PI3K-Akt and MAPK/ERK. Human postmortem and biomarker studies mainly support associations between reduced BDNF signaling, synaptic dysfunction, and AD-related pathology. In contrast, cell-based and animal studies provide mechanistic evidence that BDNF/TrkB signaling may influence amyloid precursor protein (APP) processing and neuronal resistance to A*β*-induced stress. Conversely, mechanistic studies indicate that A*β* accumulation can suppress CREB-dependent BDNF expression, disturb BDNF transport, and impair TrkB receptor function. Thus, the BDNF–A*β* relationship is better interpreted as a stage- and context-dependent pathogenic coupling rather than a simple causal loop. This review synthesizes evidence from human studies, animal models, and cellular systems to clarify how BDNF–A*β* dysregulation contributes to AD progression and to discuss the translational potential of BDNF-oriented interventions.

## 1 Introduction

Alzheimer’s disease (AD) is the most common cause of dementia in older adults and is characterized by progressive memory loss, cognitive impairment, decline in language function, and reduced capacity for activities of daily living (Scheltens et al., 2021). These symptoms may be subtle in the early stages; however, as the disease progresses, patients may develop disorientation, language dysfunction, and impaired visuospatial ability. This ultimately leads to a loss of the ability to care for themselves, making them dependent on others, which greatly affects the quality of life for both patients and their families (Jia et al., 2020). Neuropathologically, AD is characterized by the extracellular deposition of *β*-amyloid (A*β*) plaques, intracellular neurofibrillary tangles, synaptic loss, neuronal loss, and progressive atrophy of the hippocampus and cerebral cortex, leading to widespread brain dysfunction (Nelson et al., 2012).

Brain-derived neurotrophic factor (BDNF) is a key regulator of neuronal survival, synaptic maintenance, and plasticity, and increasing evidence suggests that impaired BDNF signaling is associated with AD-related synaptic dysfunction and neurodegeneration (Alqahtani et al., 2025). Cell-based and animal studies further suggest that enhancing BDNF signaling can alleviate A*β*-related pathology, preserve synaptic integrity, and improve cognitive performance in AD models (Alqahtani et al., 2025). The neuroprotective effects of BDNF involve multiple intracellular signaling pathways, particularly the Ras-MAPK/ERK and PI3K-Akt cascades, which are closely related to neuronal survival, synaptic remodeling, and resistance to A*β*-induced stress (Wu et al., 2021). In addition, reduced BDNF expression in the AD brain has been associated with increased amyloid burden and cognitive impairment (Gao et al., 2022). Conversely, A*β* pathology can impair BDNF availability and downstream signaling, potentially creating a deleterious cycle that exacerbates synaptic and neuronal dysfunction. Therefore, the current evidence supports a stage- and context-dependent pathogenic coupling between amyloid stress and neurotrophic failure rather than a simple one-directional causal relationship.

Recent studies have highlighted several aspects in this field. BDNF-centered reviews have summarized the dysregulation of BDNF–TrkB/p75NTR signaling, the imbalance between proBDNF and mature BDNF, and receptor-targeted therapeutic strategies in AD (Alqahtani et al., 2025; Harun et al., 2025). In parallel, A*β*-focused and AD therapeutic reviews have updated current understanding of amyloid biology, anti-amyloid and anti-tau strategies, multi-target drug development, and clinical trial pipelines (Azargoonjahromi, 2024; Cummings et al., 2024; Ogos et al., 2024; Sharma et al., 2025). However, these studies have generally addressed BDNF signaling, A*β* biology, or AD therapy as distinct but related topics. Less attention has been given to the temporal and mechanistic coupling between A*β* accumulation and BDNF/TrkB impairment, the stage-dependent shift between proBDNF/p75NTR and mature BDNF/TrkB signaling, and the therapeutic implications of this neurotrophic–amyloid relationship.

To define the scope of this narrative review, we searched PubMed, Web of Science, and Google Scholar for studies related to BDNF/TrkB signaling, A*β*/amyloid precursor protein (APP) processing, proBDNF/p75NTR balance, CREB-dependent transcription, Val66Met, and BDNF-oriented interventions. Recent reviews, human clinical or postmortem evidence, and mechanistic animal- or cell-based studies have been prioritized. In this review, we distinguish associative human evidence from mechanistic findings derived from animal models or cellular systems, where appropriate. This review focuses on the BDNF–A*β* axis as an integrated regulatory framework rather than a broad catalog of AD mechanisms. Specifically, we discuss how impaired BDNF/TrkB signaling may weaken synaptic resilience and influence APP processing, how A*β* accumulation may suppress BDNF expression, transport, and downstream signaling, and how this axis is linked to tau phosphorylation, neuroinflammation, oxidative stress, genetic susceptibility, and therapeutic development. By integrating mechanistic evidence with translational considerations, this review aims to clarify how BDNF–A*β* dysregulation contributes to AD progression and to identify potential intervention points for BDNF-oriented or combination therapeutic strategies.

## 2 Biological characteristics of BDNF

### 2.1 Structure and function of BDNF

Understanding the structure and function of BDNF is fundamental to elucidating its role in neural development and neurological diseases. BDNF is a key neurotrophin involved in neuronal differentiation, synaptic plasticity, survival, and repair of the central nervous system (Lu, 2003; Park and Poo, 2013). The human BDNF gene is located on chromosome 11 and encodes a precursor protein containing a signal peptide, prodomain, and a conserved mature BDNF domain. BDNF is synthesized as proBDNF, a precursor that is subsequently processed by intracellular and extracellular proteases to generate mature BDNF.

**Figure 1:**
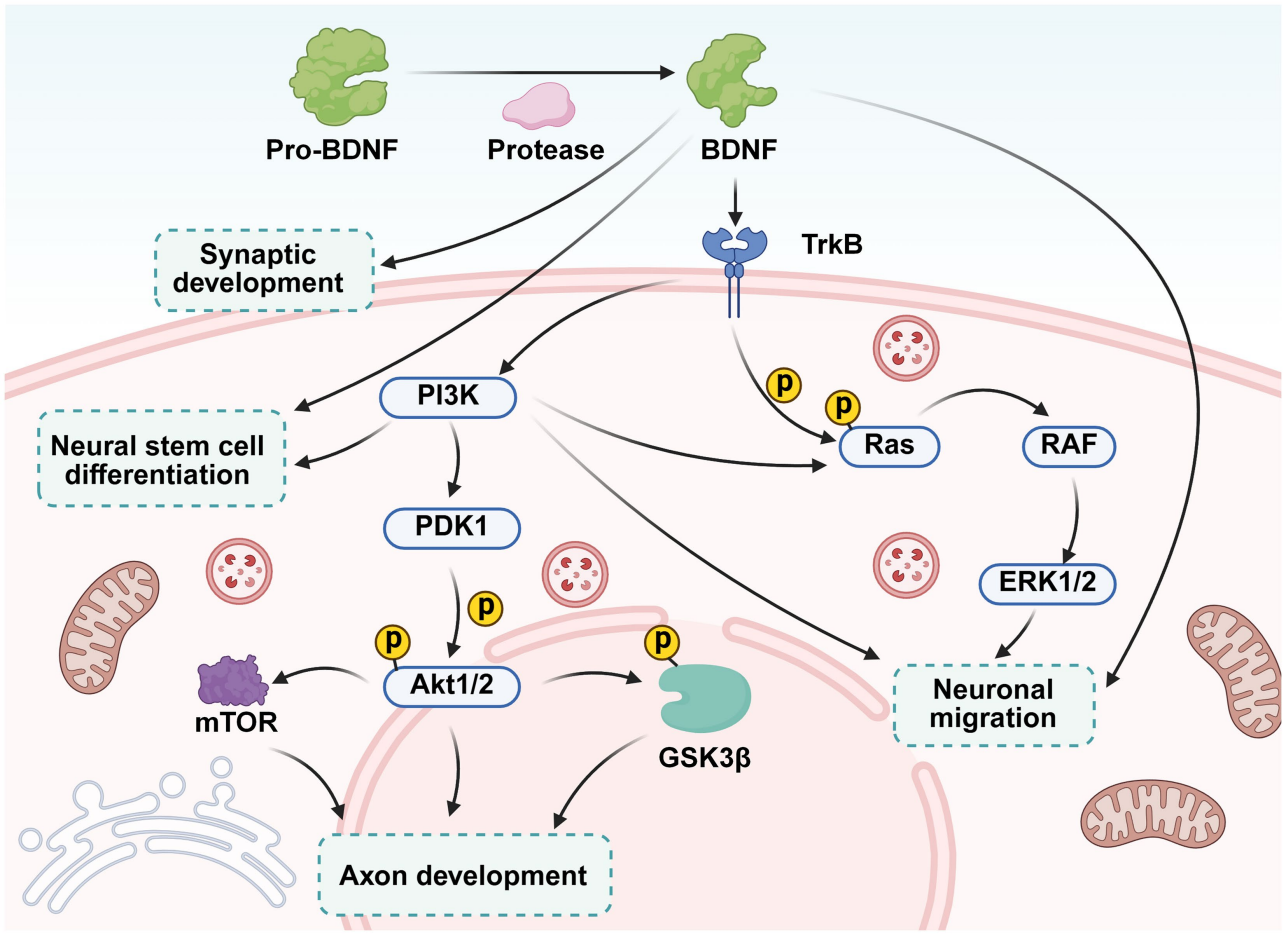
BDNF-mediated signaling pathways regulate neuronal development. Proteolytic processing converts pro-brain-derived neurotrophic factor (proBDNF) into mature BDNF, which activates tropomyosin receptor kinase B (TrkB) and triggers downstream Ras-MAPK and PI3K-Akt-mTOR signaling cascades. Ras-dependent activation of RAF and ERK1/2 contributes to cytoskeletal remodeling and neuronal migration in the developing brain. In parallel, PI3K-mediated phosphorylation of Akt promotes mTOR signaling and inhibits the phosphorylation of glycogen synthase kinase-3*β* (GSK3*β*), thereby supporting neural stem cell differentiation, axonal growth cone extension, and axon development. BDNF signaling also modulates synapse formation and stabilization, collectively facilitating neuronal maturation and plasticity.

These two forms exert distinct biological effects through differential receptor binding. Mature BDNF preferentially activates TrkB, whereas proBDNF mainly signals through the p75NTR/sortilin receptor complex, leading to divergent downstream signaling outcomes. Upon binding to TrkB, mature BDNF induces receptor dimerization and autophosphorylation, thereby activating intracellular signaling pathways including the PI3K-Akt and MAPK/ERK pathways (Park and Poo, 2013; Figure 1). These cascades collectively regulate neurogenesis, neuronal migration, synaptic maturation, neuronal survival, and activity-dependent synaptic plasticity (Lu, 2003; Park and Poo, 2013; Wu et al., 2021). In contrast, proBDNF/p75NTR signaling is more closely associated with synaptic weakening, neurite retraction, and apoptotic or stress-related responses, particularly when TrkB-mediated survival signaling is insufficient. The p75NTR/sortilin pathway has been implicated in proBDNF-induced neuronal apoptosis, impaired synaptic plasticity, inflammation, and cellular stress in neurological diseases (Teng et al., 2005; Chen et al., 2025). Therefore, BDNF function cannot be fully interpreted by total BDNF levels alone; rather, the balance between proBDNF/p75NTR and mature BDNF/TrkB signaling may determine whether BDNF-related signaling predominantly supports synaptic resilience or contributes to neuronal vulnerability under pathological conditions.

This distinction is particularly relevant in AD. Current review-level evidence and human AD tissue studies indicate that BDNF signaling dysregulation in AD and related neurodegenerative conditions involves not only reduced mature BDNF/TrkB signaling but also an altered balance between proBDNF and mature BDNF (Alqahtani et al., 2025; Mitrovic et al., 2025). In AD samples, increased proBDNF levels, sortilin expression, and proBDNF/BDNF ratios have been reported, suggesting that a shift toward proBDNF/p75NTR signaling may contribute to impaired synaptic plasticity and neuronal degeneration (Fleitas et al., 2018). Thus, the proBDNF/mature BDNF balance provides important mechanistic nuance for understanding how BDNF signaling changes during AD progression and why therapeutic approaches should aim to restore protective TrkB signaling without inadvertently enhancing p75NTR-associated pathways.

In addition to its direct effects on neuronal structures, BDNF interacts extensively with major neurotransmitter systems (Sharma et al., 2025). It modulates glutamatergic signaling by regulating the expression and function of glutamate receptors, thereby facilitating synaptic plasticity (Lu, 2003; Park and Poo, 2013). BDNF is also closely linked to the dopaminergic system, supporting the development and survival of dopaminergic neurons and influencing dopamine signaling dynamics (Park and Poo, 2013; Karkkainen et al., 2015; Zhang, X. et al., 2025; Zhang, Y. et al., 2025). Alterations in BDNF availability are associated with dysregulated DA transmission in neurodegenerative and mood disorders (Ahmad et al., 2023). Furthermore, BDNF contributes to the regulation of GABA-mediated inhibitory transmission by influencing receptor expression and synaptic strength, thereby maintaining the excitatory–inhibitory balance essential for network stability (Park and Poo, 2013; Porcher et al., 2018).

### 2.2 Genetic and epigenetic regulation of BDNF function

The Val66Met single-nucleotide polymorphism (rs6265) in the BDNF gene results in a valine-to-methionine substitution at codon 66 within the prodomain of proBDNF. As a common functional variant, Val66Met has been associated with altered intracellular trafficking and activity-dependent secretion of BDNF, thereby influencing hippocampal function, synaptic plasticity, episodic memory, and resilience to age- and dementia-related neurological dysfunction (Brown et al., 2020). In AD-related studies, Val66Met has been investigated as a potential modifier of cognitive decline and disease progression rather than as a deterministic risk factor. The Met allele has been linked to reduced resilience during brain aging and dementia, and longitudinal evidence suggests that it may contribute to the progression from subjective cognitive decline to mild cognitive impairment and AD (Bessi et al., 2020; Brown et al., 2020). Clinical and imaging studies further suggest that Val66Met may interact with the AD-related pathological burden. In prodromal AD, the Met allele has been associated with greater memory decline and hippocampal atrophy (Lim et al., 2014). In preclinical autosomal dominant AD, Val66Met may moderate the effect of amyloid pathology on memory impairment, hippocampal function, and tau-related changes (Lim et al., 2016). Recent evidence has linked Val66Met to tau hyperphosphorylation and cognitive impairment in dominantly inherited AD, supporting its potential relevance in downstream pathological processes beyond BDNF secretion alone (Lim et al., 2022). However, the overall association between Val66Met expression and susceptibility to AD remains unclear. An updated meta-analysis and high-throughput genotyping cohort study did not support a robust association between Val66Met and AD risk, including analyses stratified by sex and APOE ε4 status (Zhao et al., 2018). Therefore, Val66Met is best viewed as a context-dependent modifier of BDNF availability and AD-related vulnerability with potential interactions involving age, sex, disease stage, APOE genotype, amyloid burden, and tau pathology.

Sex may further influence BDNF-related vulnerabilities in AD. Sex-related differences have been reported in AD symptomatology, progression, biomarker profiles, risk factors, and treatment responses, supporting the need to consider sex as a biological variable in AD research (Ferretti et al., 2018). In the context of BDNF, serum BDNF levels have been reported to be reduced in patients with AD, with more pronounced changes in female patients in some cohorts, suggesting that sex-related biological factors may modulate peripheral BDNF availability and AD susceptibility (Piancatelli et al., 2022). However, sex-stratified findings regarding BDNF levels and BDNF-related genetic risks have not been fully consistent across studies. Therefore, sex should be considered in future studies on BDNF signaling, biomarker interpretation, and therapeutic response, rather than as evidence for a fixed BDNF-related risk pattern.

In addition to genetic variations and sex-related factors, epigenetic mechanisms may regulate BDNF expression in AD. Human peripheral blood and brain-based studies have associated DNA methylation in BDNF promoter regions with AD- or dementia-related phenotypes, although findings vary according to tissue source, disease stage, and analytical method (Nagata et al., 2015; Kouter et al., 2023; Tudor et al., 2025). Histone modifications, particularly histone acetylation and methylation, are also involved in activity-dependent BDNF transcription and synaptic plasticity. However, their BDNF-specific contributions to human AD remain unclear (Karpova, 2014; Singh and Paramanik, 2024). Non-coding RNAs provide another regulatory layer. For example, miR-206 has been shown to target BDNF transcripts and to regulate BDNF levels and memory-related functions in AD model systems, whereas broader reviews have indicated that multiple microRNAs can modulate BDNF expression in neuronal and disease-related contexts (Lee et al., 2012; De Assis and Murawska-Cialowicz, 2024). Together, these findings suggest that genetic, sex-related, and epigenetic factors may jointly shape BDNF availability and AD-related vulnerability, although their mechanistic links with A*β* pathology require further investigation.

### 2.3 Role of BDNF in synaptic function and AD-related vulnerability

BDNF plays a central role in development, maintenance, and activity-dependent plasticity (Lu et al., 2014). By activating TrkB, BDNF initiates intracellular signaling pathways that support neuronal growth, synapse formation, and synaptic maturation (Wang et al., 2022). Evidence from neuronal and animal studies indicates that the loss of BDNF or impaired BDNF signaling is associated with altered synaptic morphology and impaired neuronal connectivity (Yoshii and Constantine-Paton, 2010). Synaptic plasticity, which underlies learning and memory, is strongly influenced by the BDNF-mediated modulation of NMDA and AMPA receptor functions (Afonso et al., 2019; Mansvelder et al., 2019; Mohandas et al., 2025). BDNF enhances the efficacy of synaptic transmission and promotes long-term potentiation by regulating receptor trafficking, phosphorylation, and synaptic localization (Leal et al., 2015; Miranda et al., 2019). In parallel, BDNF influences activity-dependent gene expression and cooperates with other neurotrophic factors to shape postsynaptic responsiveness and neurotransmitter sensitivity (Esvald et al., 2020). Through these coordinated actions, BDNF contributes to synaptic homeostasis and supports the long-term stability and adaptability of neural circuits.

The role of BDNF in AD is closely associated with synaptic vulnerability, neuronal resilience, and cognitive function. Reduced BDNF levels and impaired BDNF/TrkB signaling have been associated with hippocampal neuronal vulnerability, synaptic dysfunction, and deficits in learning and memory (Gao et al., 2022; Alqahtani et al., 2025). Reduced BDNF expression has also been reported in key AD-affected brain regions, including the hippocampus and cerebral cortex. However, the magnitude of this change may vary according to brain region, disease stage, sample type, and detection method (Gao et al., 2022). Table 1 summarizes the representative changes in BDNF mRNA, mature BDNF, and proBDNF levels across AD-affected brain regions and disease stages. Overall, these findings indicate that BDNF dysregulation is regionally heterogeneous and may appear as early as the preclinical or MCI stages, with broader cortical and basal forebrain involvement in advanced AD. Given the established role of BDNF in mood-related neural circuits, altered BDNF signaling may also contribute to depression- or anxiety-related manifestations that often coexist with cognitive decline in AD (Martinowich et al., 2007; Martinowich and Lu, 2008). Thus, BDNF dysfunction provides an important basis for understanding AD-related synaptic and network vulnerabilities.

**Table 1:** Regional and stage-related changes in BDNF levels in AD brain tissues.

Brain region	Disease stage/sample	BDNF metric measured	Reported change	Main implication	Reference(s)
Hippocampus	Postmortem AD brain	BDNF mRNA/protein	Decreased compared with controls	Supports reduced BDNF expression in a key memory-related region vulnerable to AD pathology	Phillips et al. (1991) and Ginsberg et al. (2019)
Entorhinal cortex	Postmortem AD brain	BDNF protein	Mildly decreased or regionally altered	Suggests that BDNF changes may involve early AD-vulnerable cortical regions	Narisawa-Saito et al. (1996)
Temporal cortex	Postmortem AD brain	BDNF protein	Decreased in temporal cortical regions; changes may be region-specific	Highlights regional heterogeneity of BDNF protein reduction in AD	Connor et al. (1997) and Lee et al. (2005)
Parietal cortex	Postmortem AD brain, including advanced AD pathology	BDNF mRNA/proBDNF/BDNF protein	Decreased BDNF mRNA and protein levels, with reduced proBDNF also reported	Indicates marked cortical BDNF dysregulation in advanced AD	Holsinger et al. (2000) and Jiao et al. (2016)
Frontal/prefrontal cortex	Preclinical AD, MCI, and AD postmortem tissue	Mature BDNF, proBDNF, and BDNF transcripts	Reduced mature BDNF and proBDNF reported in MCI and AD; transcript-level changes also reported in early AD neuropathology	Suggests that frontal cortical BDNF dysregulation may begin before clinical AD and progress with disease severity	Peng et al. (2005) and Aarons et al. (2019)
Basal forebrain/nucleus basalis	Postmortem AD brain	BDNF mRNA	Decreased compared with controls	Suggests reduced local neurotrophic support for basal forebrain cholinergic neurons	Fahnestock et al. (2002)

## 3 Characteristics of amyloid-*β*

### 3.1 Generation, aggregation, and clearance of A*β*

The generation of A*β* involves a series of complex biochemical processes closely related to AD pathophysiology (Azargoonjahromi, 2024). A*β* is derived from its precursor protein, APP, through sequential proteolytic cleavage (Nelson et al., 2012). APP can be processed via two major pathways: non-amyloidogenic and amyloidogenic (Eggert et al., 2022). In the non-amyloidogenic pathway, APP is cleaved by *α*-secretase to generate soluble APPα (sAPPα) and a membrane-bound C83 fragment, thereby preventing A*β* production. In the amyloidogenic pathway, APP is first cleaved by *β*-secretase, primarily BACE1, to produce soluble APP*β* (sAPP*β*) and a membrane-bound C99 fragment. C99 is subsequently processed by the *γ*-secretase complex, resulting in the release of A*β* peptides with aggregation potential (Nelson et al., 2012). Among these peptides, A*β*40 and A*β*42 are the predominant isoforms, with A*β*42 being more prone to aggregation and more closely associated with amyloid plaque formation, making it a key species in AD pathogenesis (Yan and Wang, 2006; Chen et al., 2017).

Under physiological conditions, A*β* is continuously produced and cleared from the brain. However, in AD, the balance between A*β* generation and clearance is disrupted, leading to excessive accumulation of A*β* in both intracellular and extracellular compartments (Wu et al., 2021). A*β* clearance involves multiple mechanisms, including enzymatic degradation, cellular uptake by microglia and astrocytes, vascular transport across the blood–brain barrier (BBB), and interstitial fluid drainage through the glymphatic system. Human imaging evidence suggests that glymphatic dysfunction may precede amyloid pathology and predict amyloid deposition, neurodegeneration, and the clinical progression of AD (Huang et al., 2024). Therefore, impaired clearance is not merely a downstream consequence of A*β* accumulation but may actively contribute to the development and progression of amyloid pathology.

**Figure 2:**
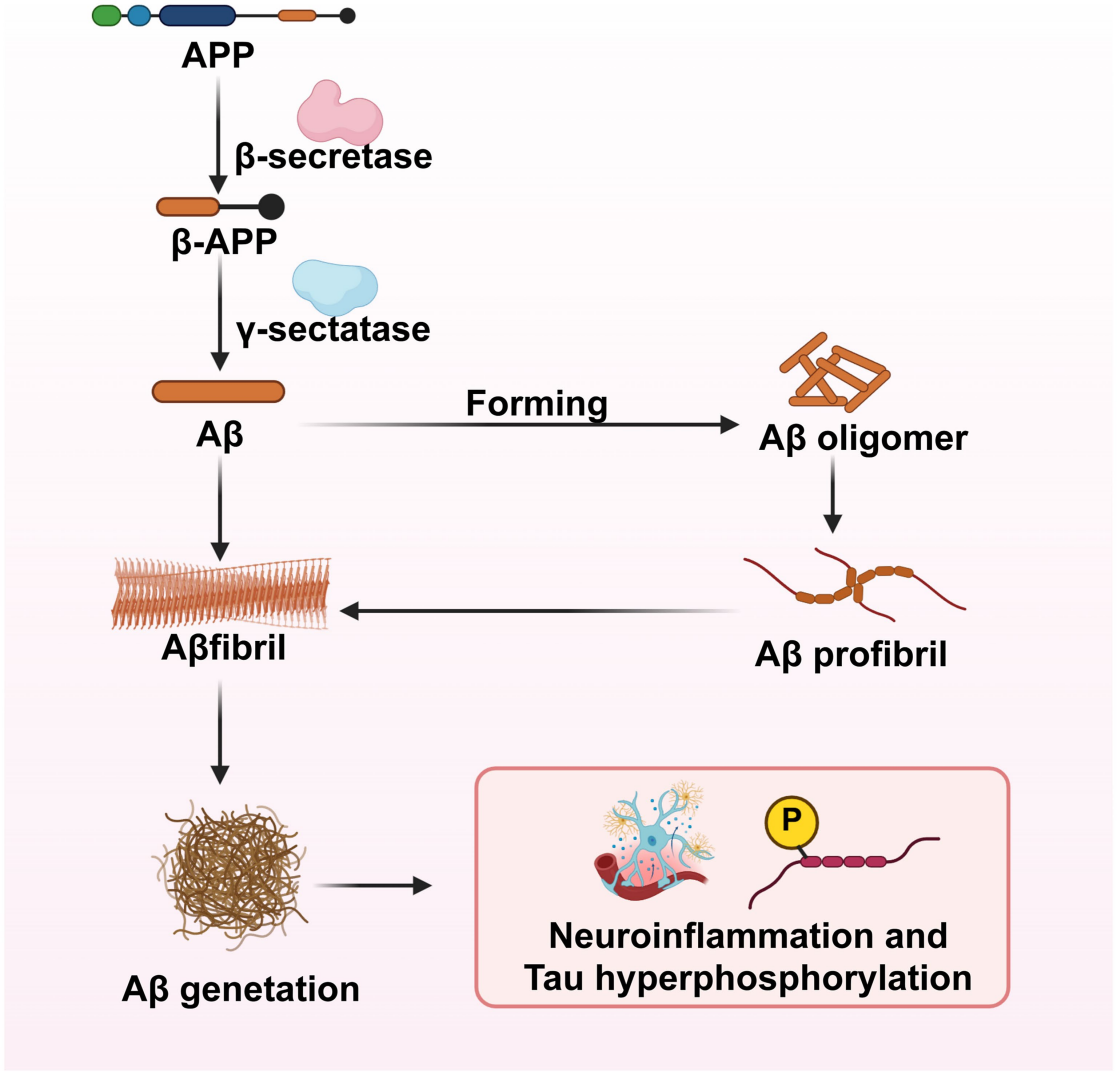
Amyloid-*β* generation, aggregation, and associated pathological cascades. Amyloid precursor protein (APP) is sequentially cleaved by *β*-secretase and *γ*-secretase complexes, producing A*β* peptides. These peptides undergo nucleation-dependent aggregation, forming soluble oligomers, protofibrils, fibrillar assemblies, and ultimately, extracellular amyloid deposits. Progressive A*β* accumulation is associated with neuroinflammatory responses and promotes tau hyperphosphorylation, contributing to neuronal dysfunction and neurodegenerative pathology.

A*β* exists in multiple aggregation states, including monomers, soluble oligomers, protofibrils, fibrils, and insoluble plaques. Monomeric A*β* peptides can nucleate and recruit additional monomers, giving rise to oligomers and amyloid fibrils (Figure 2). These fibrillar aggregates progressively accumulate and are ultimately deposited as amyloid plaques (Nelson et al., 2012; Ono and Watanabe-Nakayama, 2021). In histological sections, amyloid plaques exhibit characteristic staining properties, including Congo red birefringence under polarized light and positivity with amyloid-binding dyes, such as thioflavin stains (Ono and Watanabe-Nakayama, 2021). Importantly, these aggregated states are not biologically equivalent. Mechanistic studies have emphasized soluble A*β* oligomers as particularly relevant to early synaptic dysfunction, whereas fibrils and plaques represent more advanced aggregation states and may also serve as reservoirs for bioactive A*β* species (Azargoonjahromi, 2024).

### 3.2 Neurotoxic effects of A*β*

Evidence from cell-based and animal studies, supported by human biomarker observations, indicates that A*β* species affect neuronal function through several converging mechanisms (Azargoonjahromi, 2024). Among these forms, soluble A*β* oligomers are particularly relevant to synaptic dysfunction because they can disrupt synaptic transmission, impair long-term potentiation, alter glutamatergic receptor function, and disturb neuronal network activity. These effects contribute to cognitive impairment, including learning and memory deficits. In humans, recent biomarker evidence indicates that synaptic markers are associated with cognitive decline, even after accounting for the amyloid burden, supporting the clinical relevance of synaptic dysfunction in the AD continuum (Planalp et al., 2025). In cell and animal models, A*β* exposure has been linked to oxidative stress, mitochondrial dysfunction, calcium dyshomeostasis, and activation of apoptotic signaling pathways (Cheignon et al., 2018; Azargoonjahromi, 2024). These mechanisms can damage proteins and lipids, disrupt membrane integrity, and alter neuronal electrophysiological properties. Rather than acting independently, oxidative stress, mitochondrial impairment, and calcium dysregulation may converge to increase neuronal vulnerability.

A*β* also contributes to neuroinflammatory responses. Extracellular A*β* aggregates and soluble oligomeric species can activate microglia and astrocytes, triggering immune responses that include the release of cytokines, chemokines, complement-related molecules, and reactive oxygen species (Han et al., 2025). In addition, A*β*-associated inflammatory signaling may involve membrane microdomains and lipid raft-related inflammatory platforms, further linking A*β* accumulation to glial activation and chronic neuroinflammation (Ding et al., 2025). Although acute glial responses may contribute to A*β* clearance and tissue repair, excessive or sustained inflammation can lead to synaptic injury, neuronal dysfunction, and cell death, thereby exacerbating disease pathology. Thus, A*β* pathology should not be viewed solely as plaque deposition. Its biological impact depends on the peptide species, aggregation state, clearance efficiency, cellular targets, and disease stage. This complexity is important for understanding how A*β* disrupts BDNF/TrkB-dependent neurotrophic support in AD.

## 4 Interaction between BDNF and amyloid-*β*

**Figure 3:**
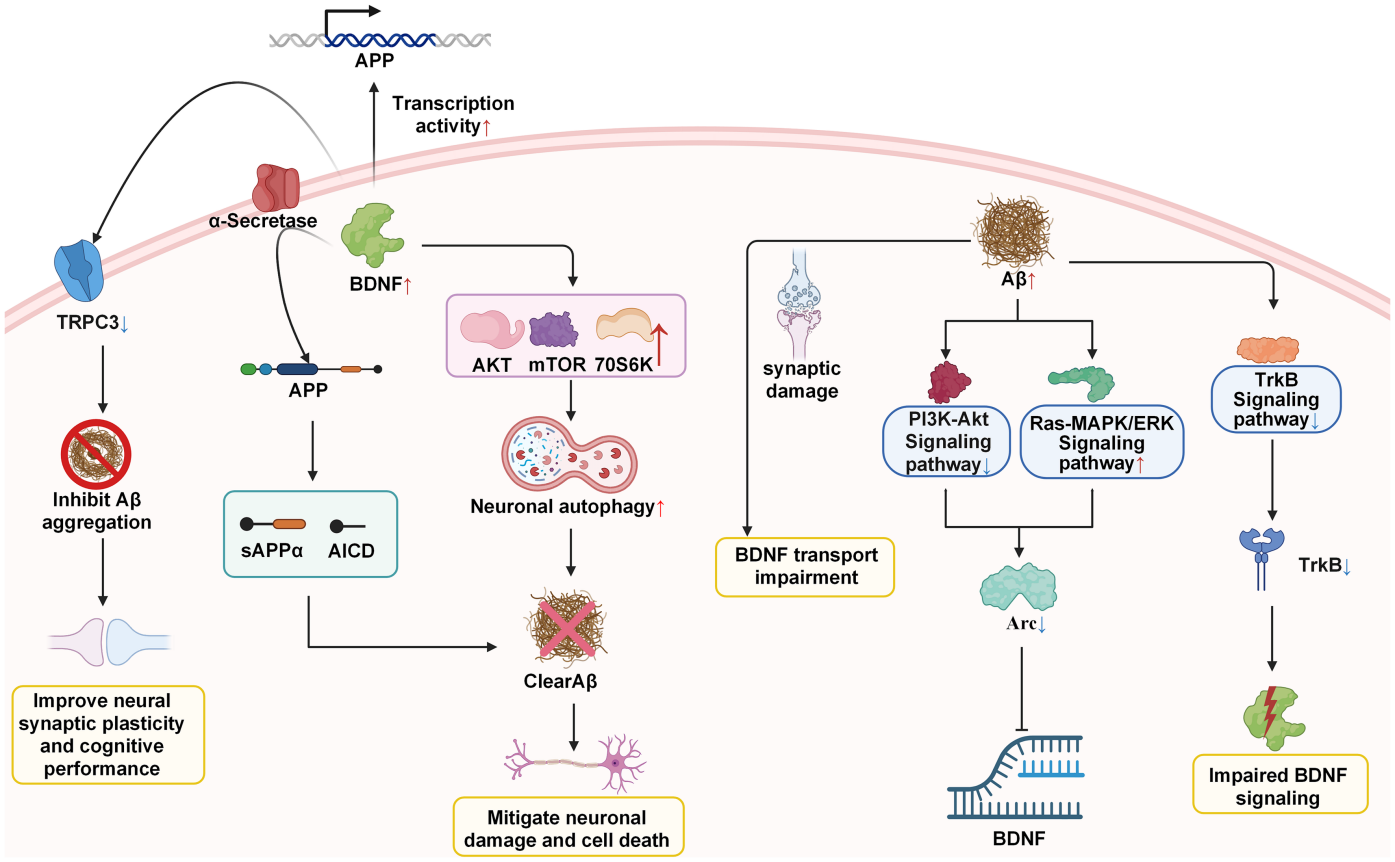
Bidirectional regulatory network between BDNF and amyloid-*β* in neurons. BDNF promotes non-amyloidogenic APP processing through *α*-secretase, generating soluble APPα (sAPPα) and APP intracellular domain (AICD), thereby facilitating A*β* clearance. BDNF also activates the Akt-mTOR-70S6K signaling pathway and enhances neuronal autophagy, further contributing to A*β* clearance and mitigating neuronal damage. In parallel, BDNF-associated TRPC3 signaling is linked to reduced A*β* aggregation, improved synaptic plasticity, and enhanced cognitive performance. Conversely, A*β* accumulation induces synaptic damage and impairs BDNF transport. Increased A*β* levels are associated with altered PI3K-Akt and Ras-MAPK/ERK signaling, reduced activity-regulated cytoskeleton-associated protein (Arc) expression, suppressed BDNF expression, and attenuated TrkB signaling. Together, these reciprocal interactions form a pathogenic feedback network that links amyloid accumulation to impaired neurotrophic support and progressive neuronal dysfunction.

The relationship between BDNF and A*β* should not be viewed as a simple circular interaction, but rather as a stage- and evidence-dependent regulatory network. A*β* accumulation can suppress BDNF expression and impair BDNF/TrkB signaling, thereby weakening neurotrophic support and increasing neuronal vulnerability. Conversely, reduced BDNF/TrkB signaling may compromise synaptic resilience and cellular homeostasis, making neurons more susceptible to A*β*-induced stress. In AD, this pathogenic coupling links amyloid burden to progressive neurotrophic failure, synaptic dysfunction, and cognitive decline (Figure 3).

### 4.1 Temporal relationship between A*β* accumulation and BDNF/TrkB impairment

A key unresolved issue is whether A*β* accumulation precedes BDNF/TrkB impairment or if early BDNF deficiency creates a permissive environment for A*β*-related pathology. Recent biological staging frameworks and longitudinal biomarker studies generally place A*β*-related abnormalities early in the AD continuum, before overt neurodegeneration and cognitive symptoms (Jack et al., 2024; Schaap et al., 2024). A longitudinal amyloid and tau positron emission tomography study of autosomal dominant AD further supports the temporal sequence in which amyloid accumulation emerges during the pre-symptomatic stage, followed by tau spreading, neurodegeneration, and cognitive decline (Sanchez et al., 2021). However, these studies did not directly determine the temporal order of BDNF/TrkB dysfunction because BDNF is not routinely included in the standard ATN biomarker framework. A*β* accumulation may impair BDNF transcription, release, and TrkB signaling, thereby converting early amyloid pathology into progressive synaptic vulnerability. Conversely, reduced BDNF availability or TrkB activity may lower neuronal resilience and aggravate memory impairment without necessarily initiating amyloid deposition. Mouse model studies have indicated that reducing TrkB signaling or BDNF availability in AD mouse models worsens memory-related deficits and AD-like signaling, whereas the effects of these manipulations on the amyloid plaque burden appear limited or model-dependent (Rantamaki et al., 2013; Devi and Ohno, 2015). Thus, the BDNF–A*β* relationship is best interpreted as a stage-dependent pathogenic amplification process: A*β* pathology may provide an early pathological stressor, while BDNF/TrkB impairment may amplify synaptic dysfunction, neuronal vulnerability, and cognitive decline.

### 4.2 Effects of BDNF on APP processing and A*β* generation

The role of BDNF in regulating APP metabolism has garnered increasing attention in recent years (Gao et al., 2022). A*β* is generated through the amyloidogenic processing of APP and plays a central role in AD pathogenesis (Nelson et al., 2012). Experimental evidence indicates that BDNF may modulate APP processing and A*β* production. In differentiated SH-SY5Y cells, BDNF treatment reduced A*β*40 and A*β*42 release. This effect was associated with enhanced *α*-secretase-dependent APP processing, increased sAPPα release, and altered distribution of ADAM10, a major α-secretase involved in APP processing; moreover, pharmacological inhibition of α-secretase attenuated the BDNF-mediated reduction in A*β* production (Nigam et al., 2017). Complementary evidence showed that acute BDNF treatment reduced sAPP*β* levels and altered BACE1 subcellular distribution in SH-SY5Y cells, suggesting that BDNF may also suppress *β*-secretase-dependent APP cleavage (Hallam et al., 2026). These findings suggest that BDNF may reduce A*β* generation by both enhancing non-amyloidogenic APP processing and limiting amyloidogenic *β*-cleavage. However, the relevance of these mechanisms to human AD requires further validation. In addition, BDNF may regulate APP processing and trafficking by modulating endoplasmic reticulum stress and autophagic pathways, thereby contributing not only to neuronal homeostasis but also to the regulation of neuroinflammatory responses (Leal et al., 2014; Numakawa and Odaka, 2021). Consistent with these observations, reduced BDNF signaling in experimental models has been associated with an increased A*β* burden and impaired cognitive performance, supporting a potential neuroprotective role for BDNF (Wu et al., 2021).

### 4.3 Interference of A*β* with BDNF transcription and TrkB signaling

A*β* suppresses BDNF expression, release, and signaling through multiple mechanisms. At the transcriptional level, A*β* may interfere with activity-dependent BDNF expression by disrupting CREB-related transcriptional regulation. The CREB-BDNF pathway is closely involved in synaptic plasticity, cognitive function, and A*β*-related neurotoxicity in AD (Amidfar et al., 2020). Cell-based evidence further indicates that A*β* decreases CREB expression and that CREB mediates A*β*-induced basal BDNF downregulation (Rosa and Fahnestock, 2015). In addition, dysregulated CRTC1-BDNF signaling in the hippocampus has been implicated in A*β* oligomer-induced long-term synaptic plasticity and memory impairment, and recent evidence suggests that CRTC1 S-nitrosylation in AD models disrupts CRTC1-CREB binding and impairs activity-dependent CREB-mediated gene expression (Yan et al., 2021; Zhang, X. et al., 2025). In addition to transcriptional suppression, A*β* accumulation may disrupt the interaction between BDNF and TrkB, inhibit TrkB activation, and promote TrkB internalization, thereby reducing the availability of functional receptors on the cell surface (Jeronimo-Santos et al., 2015; Fonseca-Gomes et al., 2024). BBB dysfunction may provide an additional route through which AD pathology compromises BDNF availability and signaling. The BBB is a regulated neurovascular interface that controls molecular exchange between circulation and the brain, and AD-related BBB breakdown involves endothelial dysfunction, altered tight-junction integrity, abnormal transporter activity, pericyte injury, and neuroinflammatory activation (Yue et al., 2024). Although BDNF can cross the BBB to some extent, peripheral BDNF measurements do not necessarily provide a direct estimate of central BDNF availability because they are influenced by the sampled blood compartment, platelet-derived BDNF, and methodological factors (Balietti et al., 2018; Gejl et al., 2019). Therefore, BBB dysfunction may disturb BDNF availability or delivery, reduce neurotrophic support in vulnerable brain regions, and weaken TrkB-dependent signaling. In parallel, A*β* has been shown to impair axonal and retrograde transport of BDNF-containing vesicles, suggesting that vascular barrier dysfunction and deficits in neuronal transport may jointly attenuate BDNF/TrkB signaling in AD (Poon et al., 2011; Seifert et al., 2016). A*β* also impairs TrkB-dependent downstream signaling pathways, including PI3K/Akt and MAPK/ERK, which are essential for neuronal survival and synaptic plasticity (Chiu et al., 2022). Moreover, A*β*-induced neuroinflammation and oxidative stress may further interfere with BDNF/TrkB signaling through the release of proinflammatory cytokines and redox imbalance (Gao et al., 2022). Synaptic dysfunction caused by A*β* deposition may also impair activity-dependent BDNF release and disrupt synaptic transmission, thereby negatively affecting TrkB-mediated biological effects (Liu et al., 2024). Collectively, these mechanisms indicate that A*β* weakens BDNF/TrkB signaling at transcriptional, receptor, transport, and downstream signaling levels, thereby contributing to synaptic dysfunction and AD progression.

### 4.4 BDNF–tau interactions and the PI3K-Akt/GSK-3*β* axis

In addition to amyloid pathology, tau hyperphosphorylation and neurofibrillary tangle formation are central features of AD, and accumulating evidence suggests that impaired BDNF/TrkB signaling can influence tau pathology through the PI3K-Akt/GSK-3*β* pathway. BDNF/TrkB signaling activates the PI3K-Akt pathway, and Akt-mediated phosphorylation of GSK-3*β* at Ser9 inhibits GSK-3*β* activity. Because GSK-3*β* is a major tau kinase, impairment of BDNF/TrkB-PI3K-Akt signaling reduces inhibitory control over GSK-3*β*, thereby facilitating tau hyperphosphorylation (Sayas and Avila, 2021; Pan et al., 2024). This mechanism provides a plausible link between A*β*-induced BDNF/TrkB dysfunction and tau pathology: A*β* accumulation or A*β*-triggered TrkB cleavage may weaken Akt activation, enhance GSK-3*β* activity, promote tau phosphorylation, and contribute to microtubule instability, impaired axonal transport, and synaptic dysfunction (Sayas and Avila, 2021; Fonseca-Gomes et al., 2024; Zhao et al., 2024). In AD models, preventing TrkB receptor cleavage has been reported to restore BDNF signaling and synaptic physiology while attenuating tau-related pathological progression (Fonseca-Gomes et al., 2024). Thus, the BDNF/TrkB-PI3K-Akt/GSK-3*β* pathway may represent a mechanistic bridge linking amyloid pathology, impaired neurotrophic signaling, and tau-related neuronal dysfunction in AD.

## 5 Therapeutic strategies targeting the BDNF–A*β* axis

### 5.1 Current AD therapeutic strategies in the context of the BDNF–A*β* axis

Current therapeutic strategies for AD include symptomatic treatments, anti-A*β* immunotherapies, anti-tau approaches, and emerging multi-target interventions (Zhang, J. et al., 2024; Cummings et al., 2025; Wu and Fuh, 2025). Symptomatic treatments, such as cholinesterase inhibitors and NMDA receptor antagonists, may provide modest cognitive or behavioral benefits but do not directly reduce A*β* burden, suppress tau pathology, or restore impaired BDNF/TrkB signaling. By contrast, anti-A*β* immunotherapies directly target amyloid pathology and currently represent the most clinically advanced disease-modifying strategy for early-stage AD. Lecanemab and donanemab have been approved for patients with AD, mild cognitive impairment, or mild dementia, but their use requires evidence of amyloid pathology and careful safety monitoring (Sims et al., 2023; van Dyck et al., 2023; Wu and Fuh, 2025). These therapies can reduce the amyloid burden and slow clinical decline. However, these studies do not directly address BDNF deficiency, TrkB dysfunction, impaired synaptic plasticity, or downstream tau- and inflammation-related neuronal injury.

From the perspective of the BDNF–A*β* axis, anti-A*β* therapy may indirectly benefit BDNF signaling by reducing A*β*-mediated suppression of BDNF expression and TrkB receptor function, as well as downstream PI3K/Akt and MAPK/ERK pathways. However, amyloid removal alone may be insufficient to fully restore synaptic resilience once BDNF/TrkB impairment, tau pathology, neuroinflammation, and neuronal network dysfunction are established. This limitation is clinically relevant because synaptic dysfunction and neuronal loss are closely related to cognitive decline and may persist despite a reduction in amyloid plaques. Therefore, BDNF-oriented approaches should be viewed not as replacements for anti-A*β* therapy, but as complementary strategies aimed at enhancing neuronal survival, synaptic repair, and functional recovery. Antitau therapies provide another important comparison. Tau pathology is more closely associated with neurodegeneration and cognitive impairment than amyloid burden in later disease stages, and current tau-targeted strategies include antibodies, aggregation inhibitors, kinase-related approaches, and therapies designed to interfere with tau propagation or post-translational modifications (Congdon et al., 2023; Zhang, J. et al., 2024). These approaches remain largely investigational, but their relevance to the BDNF–A*β* axis lies in the overlap between BDNF/TrkB signaling and tau-related pathways, particularly PI3K/Akt, GSK-3*β*, and synaptic plasticity. Therefore, enhancing BDNF/TrkB signaling may help counteract downstream tau-related synaptic and neuronal injuries, particularly when combined with amyloid- or tau-directed treatments.

Taken together, current AD therapies differ in their primary therapeutic logic. Anti-A*β* therapies target upstream amyloid burden; anti-tau approaches aim to modify downstream cytoskeletal and neurodegenerative pathology; symptomatic treatments mainly modulate neurotransmission; whereas BDNF-oriented interventions are designed to restore neurotrophic support and synaptic resilience. Therefore, BDNF-based strategies should not be viewed as replacements for amyloid- or tau-directed therapies but rather as complementary approaches that may enhance neuronal survival, synaptic repair, functional recovery, and treatment responsiveness in a stage-specific or combination-treatment context.

### 5.2 Emerging BDNF-targeted therapeutic approaches

Emerging BDNF-enhancing interventions offer new perspectives for AD treatment (Derakhshankhah et al., 2020). BDNF plays a critical role in neuroprotection, synaptic plasticity, and learning and memory; reduced BDNF levels in the AD brain are closely associated with neuronal injury and cognitive decline (Bathina and Das, 2015). Therapeutic strategies targeting this axis include approaches that enhance endogenous BDNF expression, directly or indirectly activate TrkB signaling, reduce A*β* burden to relieve its inhibitory effects on BDNF/TrkB pathways, and modulate upstream genetic, epigenetic, metabolic, or lifestyle-related regulators of BDNF expression (Wu et al., 2021; Gao et al., 2022; Alqahtani et al., 2025). The main BDNF-targeted therapeutic approaches, including gene therapy, TrkB receptor agonists, small-molecule BDNF enhancers, exercise/lifestyle interventions, and nutritional strategies, are summarized in Table 2.

**Table 2:** Overview of BDNF-targeted therapeutic approaches.

Therapeutic approach	Mechanism of action	Current stage	Key clinical trial(s) and status	Key limitations
BDNF gene therapy (Bathina and Das, 2015; Tang et al., 2025)	Increases BDNF expression in selected brain regions via AAV2-mediated gene delivery	Phase 1 clinical trial	NCT05040217: AAV2-BDNF gene therapy in early AD and MCI; Phase 1	Invasive delivery, dose control, regional targeting, and long-term safety
TrkB receptor agonists (Chiu et al., 2022)	Activates TrkB and downstream ERK/Akt signaling	Preclinical stage	No registered AD clinical trial identified	Receptor specificity, blood–brain barrier penetration, and dosing optimization
Small-molecule BDNF enhancers (Numakawa and Odaka, 2021; Chiu et al., 2022)	Enhances endogenous BDNF expression or BDNF/TrkB-related signaling	Preclinical/early clinical exploration	NCT05811000: PM012 tablet in AD; Phase 2/3	Variable efficacy, limited specificity, and uncertain disease-modifying effects
Exercise and lifestyle interventions (Choi et al., 2018; Talar et al., 2022)	Promotes endogenous BDNF production and synaptic resilience	Clinical and observational evidence	NCT02968875: Exercise training in AD with plasma BDNF outcomes	Interindividual variability, adherence, and disease-stage dependence
Nutritional strategies (Numakawa and Odaka, 2021; Gao et al., 2022)	Supports BDNF signaling through dietary bioactive compounds	Preclinical and clinical exploratory evidence	NCT01001637: Curcumin formulation in AD, Phase 2; NCT01504854: Resveratrol in AD, Phase 2 completed	Heterogeneous formulations, modest effects, and variable bioavailability

A major translational challenge is that direct BDNF supplementation has not been readily translated into a viable AD therapy. As a large neurotrophic protein, BDNF has poor pharmacokinetic properties, a short peripheral half-life, limited and unpredictable central nervous system delivery after systemic administration, and restricted diffusion within the brain parenchyma (Bathina and Das, 2015; Alqahtani et al., 2025). Effective BDNF-based therapy would require sufficient regional delivery to vulnerable circuits, sustained and spatially appropriate TrkB activation, and the avoidance of excessive ectopic or p75NTR-related off-target effects. These limitations explain why current BDNF-oriented strategies increasingly focus on targeted delivery systems and the indirect activation of BDNF/TrkB signaling. In A*β*- and tau-driven AD mouse models, hippocampal adeno-associated virus (AAV)-mediated BDNF expression induced transcriptional changes related to neuronal signaling and synaptic function, suggesting potential disease-modifying effects in experimental systems (Tang et al., 2025). AAV2-BDNF gene therapy has also entered early clinical testing in patients with biomarker-supported mild AD or AD-related MCI, highlighting the translational potential of regionally targeted BDNF restoration (Tuszynski et al., 2025). In addition to gene delivery, brain-penetrating nanocarrier systems may help overcome the delivery and pharmacokinetic limitations of BDNF, thereby improving the feasibility of BDNF-based treatments for AD (Li et al., 2026). Nevertheless, these approaches still face challenges related to invasiveness or complexity of delivery, dose control, regional specificity, long-term expression, and safety.

Small-molecule TrkB agonists, BDNF mimetics, and endogenous BDNF enhancers offer alternative approaches for overcoming the limitations of recombinant BDNF protein delivery (Numakawa and Odaka, 2021; Chiu et al., 2022). These strategies aim to activate TrkB-dependent downstream pathways, including PI3K/Akt and MAPK/ERK signaling, or increase endogenous BDNF expression through transcriptional, metabolic, or epigenetic mechanisms. Compared to direct BDNF supplementation, small molecules may offer better pharmacokinetic properties and more feasible systemic administration; however, their translational success depends on receptor specificity, BBB penetration, appropriate pathway activation, and avoidance of non-physiological or off-target signaling. Because BDNF/TrkB signaling intersects with A*β* metabolism, tau phosphorylation, neuroinflammation, and synaptic plasticity, these compounds are more plausibly positioned as components of combination therapy than as standalone disease-modifying treatments.

Among the non-pharmacological interventions, exercise deserves particular attention because it is one of the most reproducible physiological inducers of BDNF. Exercise may enhance endogenous BDNF/TrkB signaling while also improving vascular function, metabolic homeostasis, mitochondrial function, neuroinflammatory status, and synaptic plasticity, making it especially relevant to the BDNF–A*β* axis. Evidence from a systematic review and meta-analysis of AD animal models showed that exercise increases BDNF levels in the hippocampus and cortex, with significant effects reported for treadmill exercise, swimming, and voluntary wheel running (Zhang, S. et al., 2024). Exercise may also support synaptic plasticity in neurodegenerative diseases through BDNF-related and inflammation-modulating mechanisms (Farina et al., 2026). Clinical studies suggest that aerobic exercise may improve fitness, functional capacity, and quality of life in patients with AD, although its effects on circulating BDNF and cognitive outcomes are variable and may depend on exercise type, intensity, duration, disease stage, baseline physical condition, and adherence (Enette et al., 2020; Vidoni et al., 2022; Romero Garavito et al., 2024). Therefore, lifestyle interventions should consider BDNF-enhancing and multisystem strategies, rather than simple substitutes for pharmacological BDNF delivery.

Peripheral BDNF may also have translational value as a biomarker; however, its interpretation requires caution. Several studies and meta-analyses have reported altered serum or peripheral BDNF levels in patients with AD or MCI, suggesting its potential relevance for diagnosis, prognosis, and monitoring therapeutic response (Ng et al., 2019; Xie et al., 2020). However, findings are heterogeneous across disease stages and sample types. Peripheral BDNF levels are strongly influenced by age, education, occupation, blood fraction, platelet-derived BDNF, pre-analytical handling, storage conditions, and assay methodology (Balietti et al., 2018; Gejl et al., 2019; Qian et al., 2022; Kueck et al., 2025). Therefore, peripheral BDNF levels are unlikely to serve as a stand-alone diagnostic biomarker. However, BDNF may be useful as a complementary marker of neurotrophic status, disease stage, or response to BDNF-enhancing interventions when standardized sampling and analytical protocols are applied. Taken together, BDNF-targeted therapeutic strategies should be understood as complementary components of a broader AD treatment framework. Direct BDNF supplementation remains limited due to delivery and target engagement challenges. In contrast, gene therapy, brain-penetrating nanocarriers, TrkB-directed small molecules, lifestyle interventions, and biomarker-guided monitoring may provide more feasible methods to restore or evaluate BDNF/TrkB-dependent neurotrophic support.

## 6 Discussion

This review integrates evidence from human studies, animal models, and cellular systems to clarify how BDNF–A*β* dysregulation may contribute to AD-related synaptic dysfunction, neuronal vulnerability, and cognitive decline. BDNF is widely involved in neural development, synaptic plasticity, and learning and memory and is highly expressed in cognition-related brain regions, including the hippocampus, cerebral cortex, and basal forebrain, underscoring its relevance to AD-related impairment (Wang et al., 2022). Human postmortem and biomarker studies mainly support an association between reduced BDNF availability, impaired TrkB signaling, amyloid burden, synaptic dysfunction, and cognitive decline. By contrast, cell-based and animal studies provide more direct mechanistic evidence that A*β* accumulation can suppress BDNF expression, disrupt TrkB function, and impair downstream signaling pathways, including PI3K/Akt and MAPK/ERK (Jeronimo-Santos et al., 2015; Chiu et al., 2022; Fonseca-Gomes et al., 2024). Thus, the BDNF–A*β* relationship is better understood as a stage- and context-dependent pathogenic coupling rather than a simple bidirectional loop.

BDNF counteracts AD-related pathologies through several complementary mechanisms. By activating TrkB, BDNF supports neuronal survival, enhances synaptic plasticity, and improves resistance to A*β*-induced toxicity (Wang et al., 2020). Cell-based studies further indicate that BDNF can modulate APP processing and reduce A*β* generation, in part by enhancing non-amyloidogenic APP processing or by limiting amyloidogenic cleavage (Nigam et al., 2017), while BDNF/TrkB signaling may support neuronal repair, synaptic remodeling, and neuroprotective responses (Numakawa and Odaka, 2021). However, the protective role of BDNF should not be viewed as a single linear pathway. Instead, BDNF/TrkB signaling is closely linked to multiple downstream processes, including synaptic remodeling, neuroinflammation, oxidative stress, autophagy, and tau-related pathways (Sayas and Avila, 2021; Pan et al., 2024; Zhao et al., 2024). This broader regulatory network may explain why impaired BDNF signaling is closely associated with cognitive decline and disease progression in AD.

Therapeutically, the BDNF–A*β* axis offers several potential intervention points. Strategies that enhance endogenous BDNF expression, activate TrkB, reduce A*β* burden, or prevent A*β*-mediated disruption of BDNF/TrkB signaling may help preserve neuronal function (Wang et al., 2020; Chiu et al., 2022). At present, however, most BDNF-oriented approaches remain preclinical or early translational, rather than established disease-modifying therapies for AD. Selective TrkB activation is of particular interest because it may enhance neuroprotective signaling while avoiding undesirable activation of p75NTR. For example, TrkB-targeted agents such as AS86 have improved memory-related outcomes in AD model systems; however, their clinical relevance remains to be determined (Wang et al., 2020). In parallel, approaches such as gene therapy, epigenetic modulation, lifestyle interventions, and combination treatment with anti-amyloid strategies may provide complementary therapeutic avenues (Gao et al., 2022). Nevertheless, the clinical translation of BDNF-oriented therapies remains challenging because of issues related to delivery, dose control, pathway specificity, disease stage, and long-term safety.

Future studies should therefore focus on clarifying the temporal and mechanistic relationship among A*β* accumulation, BDNF/TrkB dysfunction, tau pathology, and cognitive decline. Multimodal approaches integrating molecular biomarkers, neuroimaging, electrophysiology, and genetic information may help to determine which patient subgroups are most likely to benefit from BDNF-oriented interventions. In particular, genetic factors such as the BDNF Val66Met polymorphism, together with APOE status and amyloid/tau burden, may be useful for stratified analyses in future studies (Bessi et al., 2020; Brown et al., 2020; Lim et al., 2022). Further studies are required to identify the key regulatory nodes in BDNF/TrkB signaling, optimize therapeutic regimens, and evaluate whether combining BDNF-targeted strategies with anti-amyloid or anti-tau therapies can yield more durable disease-modifying effects.

BDNF/TrkB dysregulation is also involved in other neurodegenerative diseases, including Parkinson’s disease (PD) and Huntington’s disease (HD). These disorders share several features, such as impaired synaptic plasticity, reduced neuronal resilience, and neuroinflammatory responses; however, the upstream mechanisms underlying BDNF dysfunction differ among diseases (Bathina and Das, 2015; Toader et al., 2025). In AD, A*β* accumulation is associated with reduced BDNF expression, impaired TrkB signaling, synaptic dysfunction, and inflammatory activation (Gao et al., 2022; Alqahtani et al., 2025). In PD, *α*-synuclein-related pathology can interfere with BDNF/TrkB signaling and retrograde BDNF transport, thereby contributing to dopaminergic neuronal vulnerability and impaired synaptic support (Kang et al., 2017; Miller et al., 2021). In HD, mutant huntingtin disrupts BDNF/TrkB signaling and corticostriatal BDNF transport, leading to insufficient trophic support for striatal neurons (Maloney et al., 2023; Azman and Zakaria, 2025). Thus, while BDNF dysfunction represents a shared downstream feature across these neurodegenerative diseases, disease-specific pathological proteins, including A*β* in AD, α-synuclein in PD, and mutant huntingtin in HD, affect the BDNF/TrkB axis via distinct mechanisms. This comparison highlights the common contribution of BDNF to synaptic plasticity, neuronal survival, and neuroinflammatory regulation while emphasizing the importance of disease-specific pathological contexts in the development of BDNF-oriented therapeutic strategies.
